# An Uncommon Consequence of Perforated Acute Appendicitis in Elderly Patients: Fournier’s Gangrene

**DOI:** 10.7759/cureus.66958

**Published:** 2024-08-15

**Authors:** Ali Murtada, Angelika Zielinski, Mohamed Siddig Mohamed, Hussam Khougali Mohamed, Sheik Fazal Ur Rehman

**Affiliations:** 1 General Surgery, Glan Clwyd Hospital, Rhyl, GBR; 2 Research and Development, University of Gezira, Medani, SDN; 3 General and Upper Gastrointestinal Surgery, Glan Clwyd Hospital, Rhyl, GBR; 4 General and Colorectal Surgery, Glan Clwyd Hospital, Rhyl, GBR

**Keywords:** necrotizing fasciitis, scrotal collection, intraabdominal sepsis, fournier gangrene, acute appendicitis

## Abstract

Acute appendicitis that is not diagnosed and treated promptly typically results in serious complications that raise the risk of necrotizing fasciitis, particularly in elderly patients. We present a case of a 77-year-old male, who presented to the emergency department with a clinical manifestation of Fournier's gangrene caused by acute perforated appendicitis. The patient had no symptoms or signs of an acute abdomen, and within three days he developed significant unilateral scrotal swelling and skin changes. Our case demonstrates the need to treat Fournier's gangrene as a consequence of an intra-abdominal infectious disease, particularly in elderly comorbid patients with atypical symptoms of acute appendicitis, and highlights the importance of early surgical intervention.

## Introduction

Fournier's gangrene, a rare condition with a high fatality rate of up to 67%, is a synergetic polymicrobial necrotizing fasciitis affecting the genital and perineal regions. In a few cases, Fournier's gangrene has been linked to acute appendicitis [1-3]. Even though the patient is young, healthy, immunocompetent, and has no prior history of abdominal discomfort, it is crucial to investigate a diagnosis of Fournier's gangrene due to a missed appendicitis diagnosis [2,3].

Acute appendicitis that is not diagnosed and treated promptly typically results in serious complications that raise the risk of necrotizing fasciitis, particularly in the elderly [4]. If there is anatomic diversity in the appendix, there is a chance that a CT scan will be overlooked, making it difficult to determine the underlying cause of Fournier's gangrene [3-5].

## Case presentation

A 77-year-old male presented to the emergency department with a fever, right testicular discomfort, and skin discoloration. He was not able to eat for three days due to severe nausea and vomiting. The patient also had difficulties opening his bowels since he only opened them once the day before admission with the use of laxatives; previously, he did not move his bowels for 15 days. He claimed difficulty urinating in small amounts as well as penile bleeding five days prior to admission. Examination revealed edema in the right groin as well as redness and swelling of the scrotum and penis. Upon palpation, the right testicle was more tender and enlarged than the left and sensitive to touch. No history of injuries to the abdomen or penis was accounted for.

Past medical conditions included atrial fibrillation, hypercholesterolemia, diverticulosis, previous TIA, and pulmonary emboli. He is a non-smoker and consumes around 37 units of alcohol each week. 

Upon arrival, the patient's inflammatory markers were high, including a white blood count (WBC) of 22.5, neutrophils of 19.6, and C-reactive protein (CRP) of 340 (Table 1). He was prescribed broad-spectrum antibiotics (Tazocin). A CT scan of the patient's abdomen and pelvis on the day of admission was inconclusive and showed only inflammatory processes in the right groin area. Therefore, conservative management was continued. The patient was transported to the intensive therapy unit (ITU) on the fourth day of hospitalization as he became septic and went into atrial fibrillation. He was not keen on any intervention.

**Table 1:** Blood results throughout the course of admission WBC, white blood count; CRP, C-reactive protein; eGFR, estimated glomerular filtration rate; ALP, alkaline phosphatase; ALT, alanine transaminase

	24/10/2023	29/10/2023	04/11/2023	08/11/2023	12/11/2023	24/11/2023	Reference range
WBC	22.5	25.5	15.6	14.4	11.2	7.0	4.5 to 11.0×10^9^/L
Neutrophils	19.6	24.1	11.3	10.2	8.8	3.7	1.5-8.0x10*9/L
CRP	340	183	109	49	48	9	<5 mg/L
Creatinine	107	109	99	111	90	118	60-110 ummol/L
Urea	11.4	9.3	11	6.6	5.1	8.4	2.5-7.8 mmol/L
eGFR	58	57	64	56	71	52	>90
Bilirubin	37	63	76	72	36	29	1.2 mg/dL
ALP	98	267	460	408	39	38	30-130 IU/L
ALT	20	47	78	52	45	44	7-56 units/L

**Figure 1:**
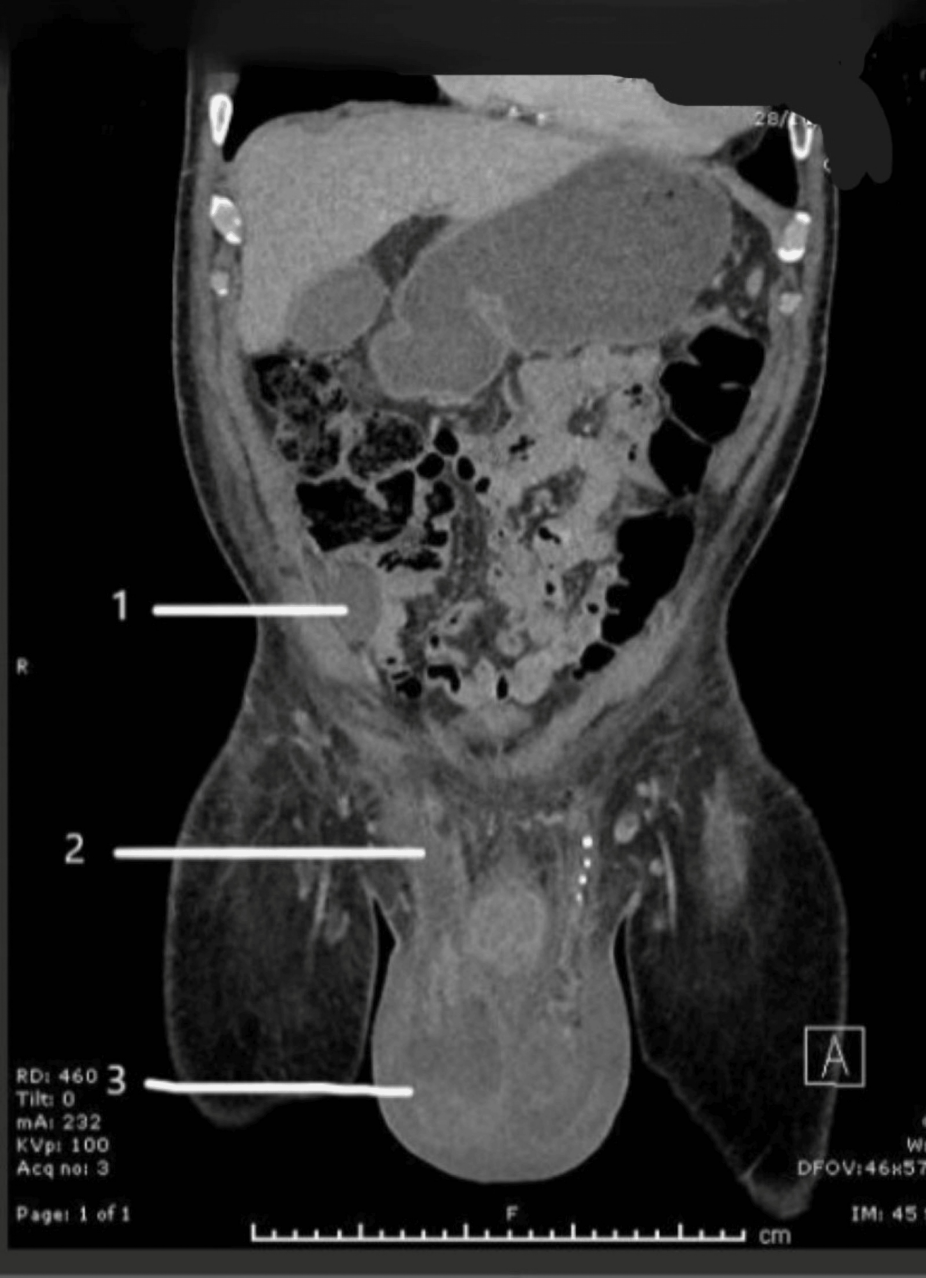
Appearance of perforated acute appendicitis (1), pus tract through the inguinal canal (2), and pus collection in right hemi-scrotum (3).

**Figure 2:**
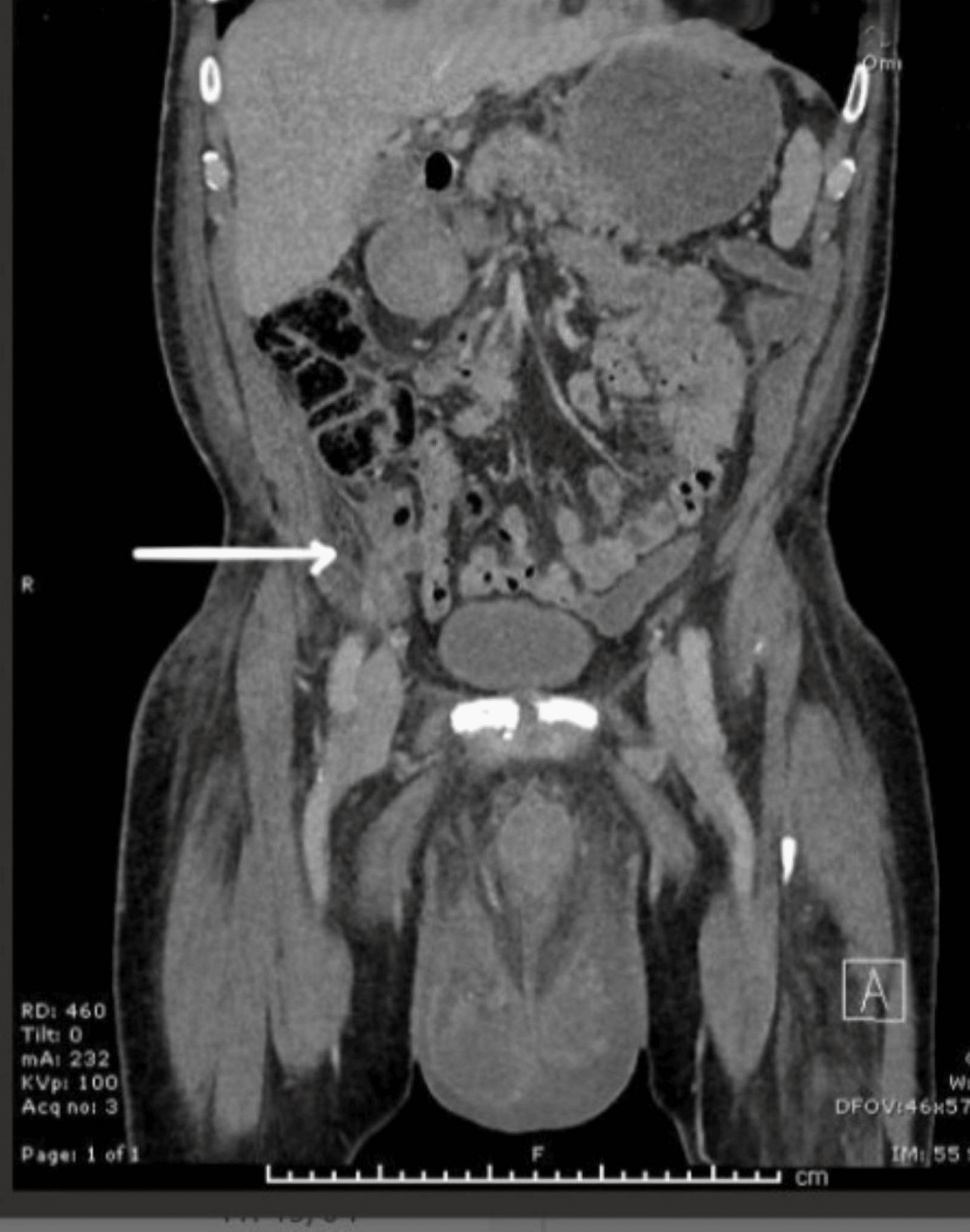
Pus collection and abscess formation through the inguinal region

**Figure 3:**
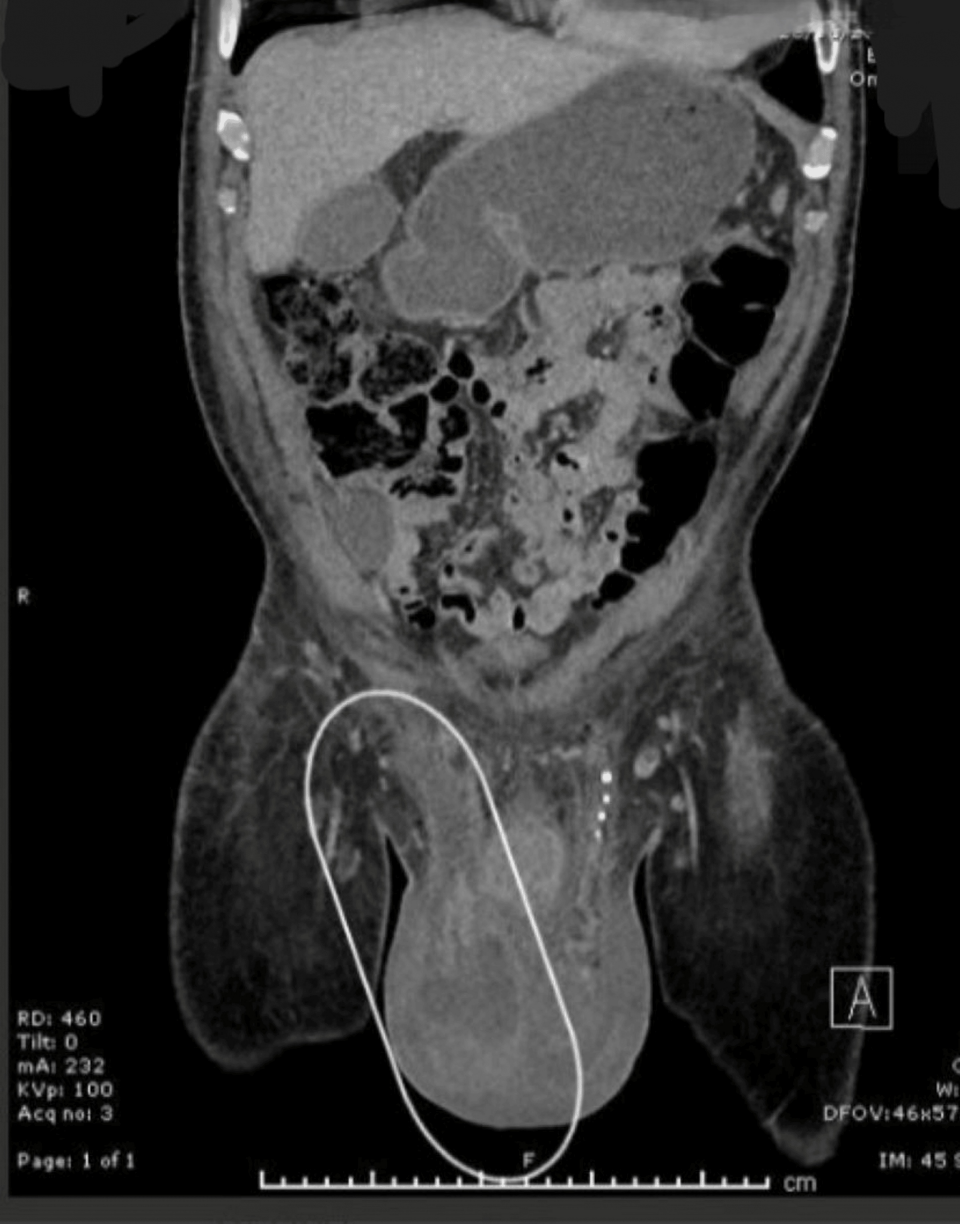
The collection tracking from the right inguinal region down to the right hemi-scrotum

A repeat CT scan on day five of admission revealed progressive inflammatory changes and minor collection in the right iliac fossa region, raising the possibility of perforated diverticulitis or appendicitis as well as significant inflammation spreading from the deep inguinal ring to the scrotal sac. The CT scan indicated a link between the right lower quadrant abscess and a possible right scrotal abscess along the fistulous tract in the inguinal canal (Figures 1, 2, 3).

**Figure 4:**
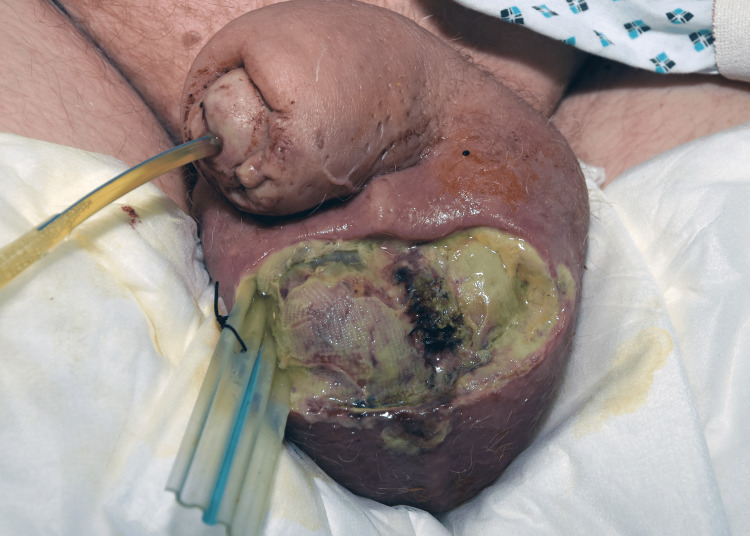
The degree of necrosis in the scrotum intraoperatively

At this time, the patient was convinced that an immediate surgical intervention was required. A combined surgery under joint care from the urology and general surgery team, including laparoscopic appendicectomy and scrotal abscess drainage and debridement, were obtained (Figure 4).

The operation was successful and there were no post-op problems. The patient stayed in the ITU for 13 days and was prescribed antibiotics (ciprofloxacin, clindamycin, and metronidazole) based on microbiology recommendations (see Figure 1). A postoperative CT scan revealed no acute intra-abdominal pathology and only normal postoperative alterations. After receiving a catheter, the patient was released home and recovered successfully. Upon follow-up, no necrosis was found in the scrotal region and all post-surgical wounds were healing well.

## Discussion

Acute appendicitis has been recognized as another cause of Fournier's gangrene, mostly related to the perforation of the retrocecal or retroperitoneal appendix with subsequent dissemination of infection into the perineal and scrotal regions, especially when there is significant pus and abscess formation close to the deep inguinal ring. This will lead to infection and pus tracking down to the scrotal region, resulting in severe Fournier's gangrene [1-4].

If the underlying cause is intra-abdominal, the diagnosis of Fournier's gangrene may be delayed and have a worse prognosis. This example highlights the vital relevance of contemplating Fournier's gangrene as a result of undiagnosed appendicitis, especially in elderly comorbid patients [5]. Furthermore, since trauma, urinary tract, and perirectal infections do not often show symptoms right away, an intra-abdominal source should be taken into account when there is a clinical suspicion of Fournier's gangrene and the underlying cause is unknown [6].

When the symptoms of acute appendicitis are atypical, vague, or unusual, such as in our patient, good conservative management and early broad-spectrum antibiotics are very important to calm the infection down [6,7]. Since necrotizing fasciitis frequently manifests as moderate erythema in the skin, similar to ordinary cellulitis, early identification may be challenging [7]. It quickly spreads along fascial planes, producing extreme toxicity and a dismal prognosis [4].

The CT scan is still the preferred method of examination for diagnosing intra-abdominal sepsis-related Fournier's gangrene, even if it was inconclusive in the early stages of our case. It is still quite sensitive [8,9]. Early surgical intervention is necessary in such a case for good outcomes and prognosis. Radical debridement of the necrotic area with a good course of postoperative antibiotics can minimize the length of hospital stay and facilitate postoperative recovery [8-10].

## Conclusions

It is critical to rule out the potential of perforated appendicitis anytime there is evidence of Fournier's gangrene, even with unusual symptoms of acute appendicitis. Our case highlights the need to treat Fournier's gangrene as a consequence of an intra-abdominal infected process, especially in elderly comorbid patients presenting with atypical acute appendicitis symptoms. It also highlights the importance of prompt surgical intervention and the administration of broad-spectrum antibiotics in the management of such cases.
